# Environmental Features Supporting Non-transportation Walking in Older Dwellers in a Hilly Neighbourhood

**DOI:** 10.1093/geroni/igab046.2355

**Published:** 2021-12-17

**Authors:** Ryoichi Nitanai, Ryogo Ogino, Daisuke Umemoto, Jun Goto, Junichiro Okata

**Affiliations:** 1 the University of Tokyo, Tokyo, Tokyo, Japan; 2 Saga University, Saga, Saga, Japan; 3 The University of Tokyo, Bunkyo-ku, Tokyo, Japan; 4 Tokai University, Hiratsuka, Kanagawa, Japan; 5 Meiji University, Chiyoda-ku, Tokyo, Japan

## Abstract

Walking is the basic mode of transportation; however, it is also considered as a recreational and physical activity. For elderly people, non-transportation walking (NTW) is necessary to maintain a good health; thus, irrespective of topography, living in an environment conducive to NTW is essential for the ageing community. This case study explores the features of the physical environment supporting NTW in older people, living in a hilly Japanese neighbourhood. We conducted semi-structured interviews with 23 older participants, with 6 being in their seventies, 13 in their eighties, and 4 in their nineties. We investigated the destinations and routes of their outings for a week, as well as their perception of walkability. Thereafter, we analysed the location of the NTW and the rationale behind the location choice. Consequently, four groups of people were identified based on their walking location: those who walked within a 1 km radius zone (N=6), those who walked outside the zone (N=8), those who walked both within and outside the zone (N=3), and those who did not walk (N=6). Moreover, each group had varied expectations regarding the physical environment, which is determined by their motivations and physical conditions, relating to the land use of the location of NTW. This implies the necessity of target identification and a suitable environmental approach for the target to promote NTW among older people in a hilly residential neighbourhood, such as improving comfort and connectedness by installing rest spots for the within-and-outside the zone walking group.

